# Foreign Body in the Left Bronchus

**Published:** 1897-08

**Authors:** George A. Himmelsbach

**Affiliations:** Buffalo, N. Y.; Assistant attending physician Buffalo General Hospital, attending physician Erie County Hospital


### FOREIGN BODY IN THE LEFT BRONCHUS.

BY GEORGE A. HIMMELSBACH, M. D., Buffalo, N. Y.
Assistant attending physician Buffalo General Hospital, attending physician Erie County Hospital.

OCTOBER 2, 1896, Anna B., aged 21 years, consulted me, complaining of periodical attacks of dyspnea and violent paroxysms of coughing and wheezing. Her family history was clear as regards asthma, tuberculosis or any pulmonary disease. A careful and searching analysis for a cause was made, but with no success. The urine was scrutinised. I looked carefully for some external or internal reflex cause, but was still in the dark. A careful physical examination of the chest was made and much to my surprise I found the disturbance to be unilateral—located in the left lung, where all the physical signs of bronchitis and asthma were present. I was now more mystified than ever. I questioned her closely and learned that, six weeks before, she consulted a dentist and had five back teeth extracted. These were removed under the influence of gas. I tried to convince myself that the gas in this instance irritated the bronchial mucosa, yet this did not explain the one-sidedness of the malady.

I began to treat the case empirically. The emunctories were kept active. She was given small doses of potassium iodide and semi-weekly I gave her a half hour's treatment with the Underwood inspirator, consisting of inhalations of air raised to a high degree of temperature and medicated with eucalyptol, naphthaline, menthol, oil of tar and the like, and occasionally medicated heated oxygen was administered. This plan of treatment was kept up for some weeks. She would seem much improved and benefited at the time of treatment, but would invariably return with the report "no better," and physical examination of her chest from time to time showed no sign of improvement.

On December 18th she consulted me the last time and she now informs me that the week subsequent to this last treatment she was worse than ever, saying that at times she would strangle and gasp for air; her cough was severe and sometimes she would raise blood; her dyspnea was most distressing.

On Tuesday, January 5, 1897, she had to do the family washing. She was obliged to ascend two flights of stairs to the attic to hang up the clothes for drying. While in the act of climbing the stairs, she was suddenly seized with a paroxysm of coughing and dyspnea almost to the point of suffocation. However, she managed to reach the top floor, where she was again seized with a paroxysm of coughing and suddenly, and with considerable force, a mass dropped from her mouth to the floor with a thud. Her curiosity was aroused and she examined the sputum, when to her surprise she found one of her molar teeth, which had been extracted fully six months before. She then examined the tooth more carefully and, singularly enough, she found a small pledget of cotton packed in a cavity of the tooth, which had never become detached or loosened. It seems for some time before extraction the dentist endeavored to save the tooth and had treated it several times with the view of filling it.

My patient experienced immediate relief the instant the foreign body was removed. Dyspnea never recurred and the bronchial cough lasted about one week, when finally it disappeared. I have since examined the lungs and can find no trace of her former trouble.

I think it is plainly evident from the foregoing history, that last August, while this girl was under the influence of the anesthetic, one of the teeth escaped the attention of the operator and found its way into the larynx, thence through the trachea to the left bronchus. Whether it entered the left bronchus or simply leaned a little more to that side at the division ofthe trachea I am unable to say. What surprises me most is, that my patient did not develop an infectious pneumonia or a pulmonary abscess, teeth being proverbially alive with orgauisms and particularly carious teeth.

In conclusion, I would like to call attention to the use of the Röntgen rays in obscure cases like the one here related. It is plain to all, that had this new and invaluable device been used six months before, the tooth and its exact location would have been found and my efforts directed towards relief of this foreign body. Besides, my patient's misery and wretchedness would have been materially curtailed.

137 W. Tupper Street.

